# Global Noncoding microRNA Profiling in Mice Infected with Partial Human Mouth Microbes (PAHMM) Using an Ecological Time-Sequential Polybacterial Periodontal Infection (ETSPPI) Model Reveals Sex-Specific Differential microRNA Expression

**DOI:** 10.3390/ijms23095107

**Published:** 2022-05-04

**Authors:** Chairmandurai Aravindraja, Matteen R. Kashef, Krishna Mukesh Vekariya, Ravi K. Ghanta, Shama Karanth, Edward K. L. Chan, Lakshmyya Kesavalu

**Affiliations:** 1 Department of Periodontology, College of Dentistry, University of Florida, Gainesville, FL 32610, USA; achairmandurai@dental.ufl.edu (C.A.); matteenkashef@ufl.edu (M.R.K.); kvekariya@ufl.edu (K.M.V.); 2 Michael E. DeBakey Department of Surgery, Baylor College of Medicine, Houston, TX 77030, USA; ravi.ghanta@bcm.edu; 3 Cancer Control and Population Sciences Program, Institute on Aging, University of Florida, Gainesville, FL 32610, USA; shama.karanth@ufl.edu; 4 Department of Oral Biology, College of Dentistry, University of Florida, Gainesville, FL 32610, USA; echan@dental.ufl.edu

**Keywords:** periodontal disease, miRNAs, NanoString analysis, partial human mouth microbes, ecological time-sequential polybacterial periodontal infection

## Abstract

Periodontitis (PD) is a polymicrobial dysbiotic immuno-inflammatory disease. It is more prevalent in males and has poorly understood pathogenic molecular mechanisms. Our primary objective was to characterize alterations in sex-specific microRNA (miRNA, miR) after periodontal bacterial infection. Using partial human mouth microbes (PAHMM) (*Streptococcus gordonii*, *Fusobacterium nucleatum*, *Porphyromonas gingivalis*, *Treponema denticola,* and *Tannerella forsythia*) in an ecological time-sequential polybacterial periodontal infection (ETSPPI) mouse model, we evaluated differential mandibular miRNA profiles by using high-throughput Nanostring nCounter^®^ miRNA expression panels. All PAHMM mice showed bacterial colonization (100%) in the gingival surface, an increase in alveolar bone resorption (*p* < 0.0001), and the induction of a specific immunoglobin G antibody immune response (*p* < 0.001). Sex-specific differences in distal organ bacterial dissemination were observed in the heart (82% male vs. 28% female) and lungs (2% male vs. 68% female). Moreover, sex-specific differential expression (DE) of miRNA was identified in PAHMM mice. Out of 378 differentially expressed miRNAs, we identified seven miRNAs (miR-9, miR-148a, miR-669a, miR-199a-3p, miR-1274a, miR-377, and miR-690) in both sexes that may be implicated in the pathogenesis of periodontitis. A strong relationship was found between male-specific miR-377 upregulation and bacterial dissemination to the heart. This study demonstrates sex-specific differences in bacterial dissemination and in miRNA differential expression. A novel PAHMM mouse and ETSPPI model that replicates human pathobiology can be used to identify miRNA biomarkers in periodontitis.

## 1. Introduction

MicroRNAs (miRNAs) are small, noncoding, regulatory RNAs that play a major role in immune inflammation-mediated periodontal disease (PD). miRNAs are ~22 nucleotides in length and regulate the expression of many messenger RNAs (mRNAs) by directly binding to their 3′ untranslated regions [1]. Prior in vitro and in vivo studies from our laboratory [2] and others have shown that miR-146a [1,3,4], miR-30e, miR-106b [5], and miR-155 [6,7,8] are overexpressed in diseased gingival tissues, salivary glands, and gingival crevicular fluid (GCF), indicating their involvement in bacterial infection-induced PD pathogenesis. Both innate and adaptive immune systems are affected by miRNA; therefore, studying the role of miRNA in inflammation-mediated periodontitis aids in developing prognostic/diagnostic biomarkers. PD affects > 20% of the world’s population, and its prevalence varies between men and women [9]. These observed differences between sexes are likely due to the influence of sex hormones on periodontal inflammation and alveolar bone metabolism [10], sexual dimorphism in periapical inflammation and alveolar bone loss, differences in bone turnover and lymphocyte recruitment [11], and differences in the jaw as it grows, develops abnormalities, and ages [12,13]. Furthermore, men have a stronger inflammatory response to bacterial infection and greater osteoclast-driven alveolar bone resorption (ABR) than women [14]. Moreover, a recent study also reported sex-specific differences in the oral microbiome in patients with severe periodontitis [15].

Prior research has reported the differential expression (DE) of specific miRNAs in a PD animal model [2]. However, this study is the first to evaluate the DE of miRNA in a more clinically relevant ETSPPI mouse model stratified by sex and using an unbiased global high-throughput miRNA analysis. No single microbe is responsible for the progression of periodontitis in humans; microbes are linked with synergistic (physiological, nutritional, and biochemical) sequential interspecies and interkingdom interactions of several microbes, including the early, intermediate, and late bacterial colonizers of the periodontium. Several studies report that *P. gingivalis* (*Pg*), *T. forsythia* (*Tf*)*, T. denticola* (*Td*), and *F. nucleatum* (*Fn*) (coaggregating bacteria) are identified more frequently and in higher numbers in adults with periodontitis than in healthy individuals, and they are also positively correlated with pocket depth and bleeding on probing, which are measurements of periodontal tissue destruction [16]. *Streptococcus gordonii* (*Sg*), an early colonizer of dental plaque, coaggregates with many oral species, including intermediate colonizer *F. nucleatum* [17] and late colonizer *P. gingivalis* [18]. The coadhesion of *P. gingivalis* to *S. gordonii* is a key point in the pathogenesis of PD [18,19]. *F. nucleatum* strongly bridges Gram-positive (Gram+) and Gram-negative (Gram−) bacteria and assists other periodontal bacteria through the enhanced adhesion and invasion of host cells [20,21]. Accordingly, *Sg*+*Fn*+*Pg/Td/Tf* PAHMM and ETSPPI have a synergistic pathogenicity. 

Here, we designed and characterized an ETSPPI mouse model using five bacteria *Sg*+*Fn*+*Pg/Td/Tf* in C57BL/6J mice (male and female). In addition, we used high-throughput miRNA expression profiling, a recent innovation, using the Nanostring nCounter^®^ system to study the global DE of miRNA in PAHMM-infected mice. We identified several significant sex-specific alterations in gingival tissue miRNAs after ETSPPI. Five of the miRNAs, namely miR-377, miR-690, miR-1274a, miR-669a, and miR-199A-3p, are reported here for the first time in mice. The other two miRNAs, miR-9 and miR-148a, have been shown to be expressed in inflamed human gingival tissues [1,6,22]. We also report bacterial colonization in the oral surface of mice after each reinfection and measure horizontal ABR and the immunoglobulin G (IgG) immune response against the bacteria to indicate PD outcomes in this newly developed ETSPPI mouse model. Our study provides insight into the sex-specific miRNA expression profile of bacterial infection. In addition, this study highlights the basis and rationale for including both sexes as potential biomarkers in the study of global miRNA expression patterns in periodontitis.

## 2. Results

### 2.1. ETSPPI-Induced PAHMM Colonization in C57BL/6J Mice

Nine-week-old male and female mice were randomly divided into groups for ETSPPI (*n* = 10) and sham-infection (*n* = 10) (Table 1). Analysis of oral gingival plaque samples from each mouse after bacterial infection [colony polymerase chain reaction (PCR)] showed the presence of bacterial 16S rRNA gene amplicons in agarose gel electrophoresis. Both sexes of mice infected with the early ecological colonizer *S. gordonii* showed the presence of *S. gordonii*-specific 16S rRNA gene amplicons (100%) after the first infection cycle, indicating that all mice were colonized with Gram+ *S. gordonii*. Previous studies showed that this is a critical ecological event for the adherence or coaggregation of the late colonizer bacterium *P. gingivalis* with the initial colonizer *S. gordonii* supragingival, and *P. gingivalis* enters the subgingival cavity to multiply [18,19]. However, the intermediate colonizer *F. nucleatum* (found in 50% of the male mice and 70% of the female mice) was colonized after its first infection cycle, and 100% colonization was observed after the second infection cycle. Similarly, *F. nucleatum* colonization is critical because it bridges with numerous Gram+ supragingival and Gram− subgingival late colonizers (e.g., *P. gingivalis*, *T. denticola*, and *T. forsythia*). Subsequently, after 3 weeks of infection, polymicrobial infection with *P. gingivalis*, *T. denticola*, and *T. forsythia* showed colonization on the gingival surfaces of all mice (100%) (Table 2). None of the sham-infected (male or female) mice were positive at any point for genomic DNA of any of the five bacteria analyzed. These results confirmed the colonization of the five oral bacteria and the development of the PAHMM mouse model.

### 2.2. ETSPPI Increased Alveolar Bone Resorption, IgG Antibody Response, and Dissemination of Bacteria to Distal Organs

**Figure 1:**
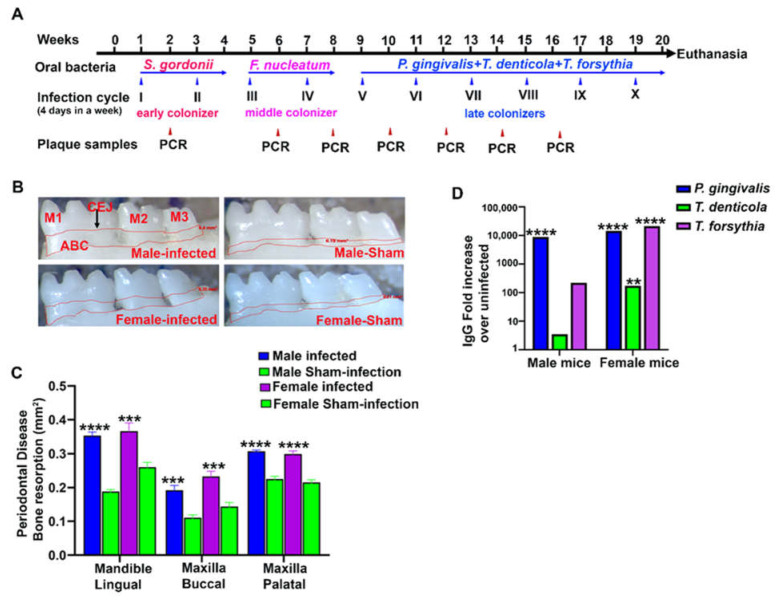
Gingival infection of periodontal bacteria significantly induced alveolar bone resorption (ABR) and elicited an IgG immune response in male and female mice. (**A**) Schematic diagram of the experimental design depicting the ecological time-sequential polybacterial periodontal infection (ETSPPI) model with early, middle, and late colonizers (4 days per week on every alternate week), plaque sampling for PCR and euthanasia. (**B**) Representative images showing horizontal ABR (mandible lingual view) of ETSPPI mice and sham-infected mice with the area of bone resorption outlined from the alveolar bone crest (ABC) to the cementoenamel junction (CEJ). (**C**) Morphometric analysis of the mandible and maxillary ABR in mice. A significant increase in ABR was observed in bacteria-infected mice compared to sham-infected mice (****, *p* < 0.0001; ***, adjusted *p*-value = 0.001; ordinary one-way ANOVA). There was no significant difference in ABR between male and female ETSPPI mice. (**D**) Serum IgG antibody levels in male and female mice. In female mice, a significantly higher IgG immune response was observed to *P. gingivalis* (> 10^4^-fold; ****, *p* < 0.001), *T. forsythia* (> 10^4^-fold; ****, *p* < 0.001), and *T. denticola* (> 10^2^-fold; **, *p* < 0.01), whereas a significantly higher IgG immune response was observed only against *P. gingivalis* (> 10^4^-fold; ****, *p* < 0.001) in male mice; ordinary one-way ANOVA). Data points and error bars are mean ± SEM (*n* = 9–10).

Periodontitis was examined using morphometry by measuring the horizontal ABR. Both male and female mice infected with PAHMM had a higher ABR in the mandible (lingual) (*p* < 0.0001 for male mice; adjusted *p*-value = 0.0001 for female mice) and maxilla (palatal) (*p* < 0.0001 for male and female mice). Similarly, a higher ABR was also observed in the maxilla (buccal) in both male and female mice (adjusted *p*-value = 0.0001; Figure 1B,C). Accordingly, there were no differences in ABR (periodontitis outcome measure) between male and female ETSPPI mice. Serum from all mice was evaluated for the humoral IgG immune response against the formalin-killed (FK) whole-cell antigens of *S. gordonii*, *F. nucleatum*, *P. gingivalis*, *T. denticola,* and *T. forsythia*. The IgG immune response was higher in female mice against *P. gingivalis* (> 10^4^-fold; *p* < 0.001), *T. forsythia* (> 10^4^-fold; *p* < 0.001), and *T. denticola* (> 10^2^-fold; *p* < 0.01), whereas a higher IgG immune response was observed only against *P. gingivalis* (> 10^4^-fold; *p* < 0.001) in male mice (Figure 1D). No IgG antibody response was observed against *S. gordonii* or *F. nucleatum* in either male or female infected mice. Bacteria-specific genomic DNA from all five oral microbes was identified in the heart after ETSPPI (Table 3) using PCR (*S. gordonii* DNA in male (9/10), female (4/10); *F. nucleatum* DNA in male (9/10), female (3/10); *P. gingivalis* DNA in male (4/10), female (2/10); *T. denticola* DNA in male (10/10), female (0/10); *T. forsythia* DNA in male (9/10), female (5/10)). This finding suggests a synergistic interaction leading to the physiological colonization/infection of the gingival epithelium and the intravascular dissemination of bacteria to the distal organs. All five bacteria disseminated from the oral gingival surface to the heart, representing their invasive potential. Specifically, intravascular dissemination was higher in the heart tissue in males than females, and this was significant for *F. nucleatum* and *T. denticola* (* *p* < 0.05, Fisher’s exact test). Furthermore, there was significantly higher dissemination of all bacteria except *T. denticola* in the lungs of females than males (* *p* < 0.05, Fisher’s exact test) (Table 3). *S. gordonii* DNA was present in other internal organs, including the lungs, kidney, and liver. DNA of all five bacteria was more prevalent in the lungs of female mice than male (Table 3).

### 2.3. Nanostring Analysis of miRNAs in Bacteria-Infected Mandibles

**Figure 2:**
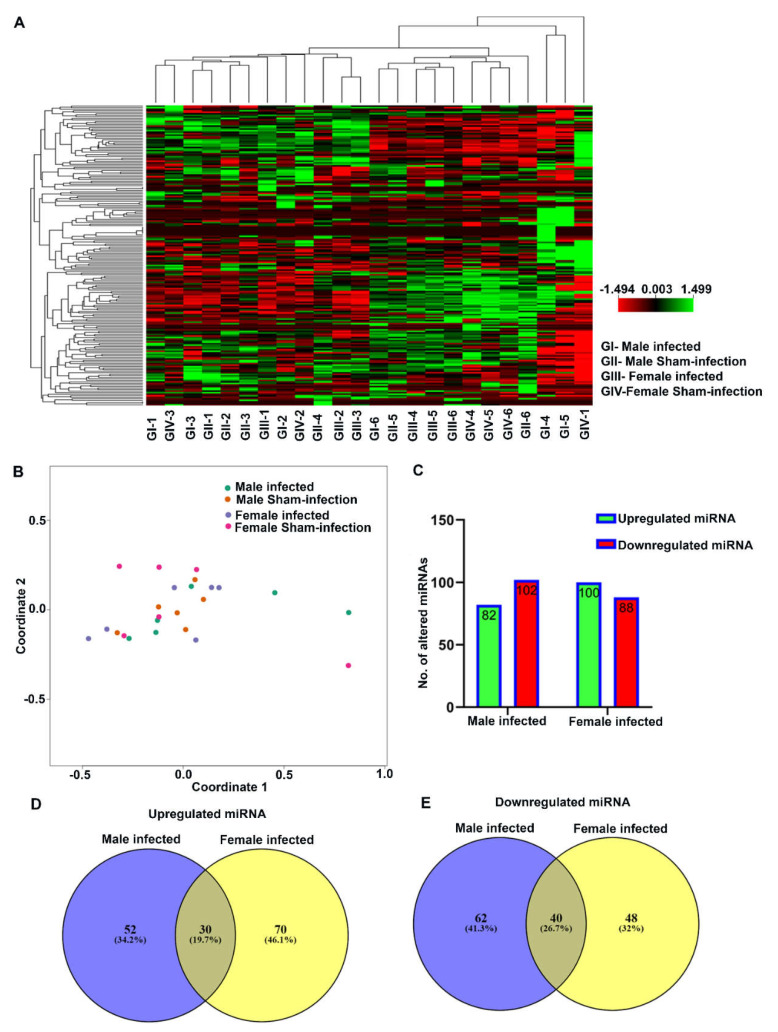
miRNA profiling in the mandibles of ecological time-sequential polybacterial periodontal infection (ETSPPI) compared to sham-infected mice and the differential expression (DE) of miRNAs in male and female mice infected with partial human mouth microbes (PAHMM). (**A**) Heat map representation of miRNAs upregulated (green) and downregulated (red) in bacterial and sham-infected mouse mandibles. Rows indicate the DE of miRNAs (contains the geometric mean of expression levels of all samples from each group) and columns represent the profiled samples (*n* = 6) in each group. (**B**) Multidimensional scaling plot shows sample clustering. Samples with similar miRNA expressions are clustered together, and dissimilar miRNA expressions are segregated. (**C**) Number of upregulated and downregulated miRNAs in both male and female infected mice compared to control mice. (**D**) Upregulated miRNAs between male and female mice infected with PAHMM compared to sham controls. Most of the miRNAs expressed (52 in male and 70 in female) were unique to the group, and 30 miRNAs were commonly upregulated in both male and female mice. (**E**) Downregulated miRNAs between male and female mice infected with PAHMM compared to controls. Most of the miRNAs expressed (62 in male and 48 in female) were unique to the group, and 40 miRNAs were commonly downregulated in both male and female mice.

The nucleic acid purity (260/280 and 260/230 ratio) of total RNA isolated from both bacteria-infected and sham-infected (both sexes) mouse mandibles (*n* = 6) was above 2, which was high enough to proceed with sample preparation in the nCounter miRNA assay (Table S1). The expression levels of spike-ins and positive and negative controls were similar in all the samples that showed ligation. Hybridization was efficient in all the mandible samples during sample preparation (Figure S1A). A heatmap representation showed the DE of miRNAs between male and female mice in bacteria-infected and sham-infected groups (Figure 2A). The multidimensional scaling plot also showed sample clustering and the similarity of miRNA expression in each sample. It is one of the most unique graphical representations of data to show the relationship between the samples. Samples with similar miRNA expressions were clustered together, and samples with dissimilar miRNA expression were segregated. PAHMM and ETSPPI male and female mice showed the DE of miRNA between samples (Figure 2B).

### 2.4. Differentially Expressed miRNAs

**Figure 3:**
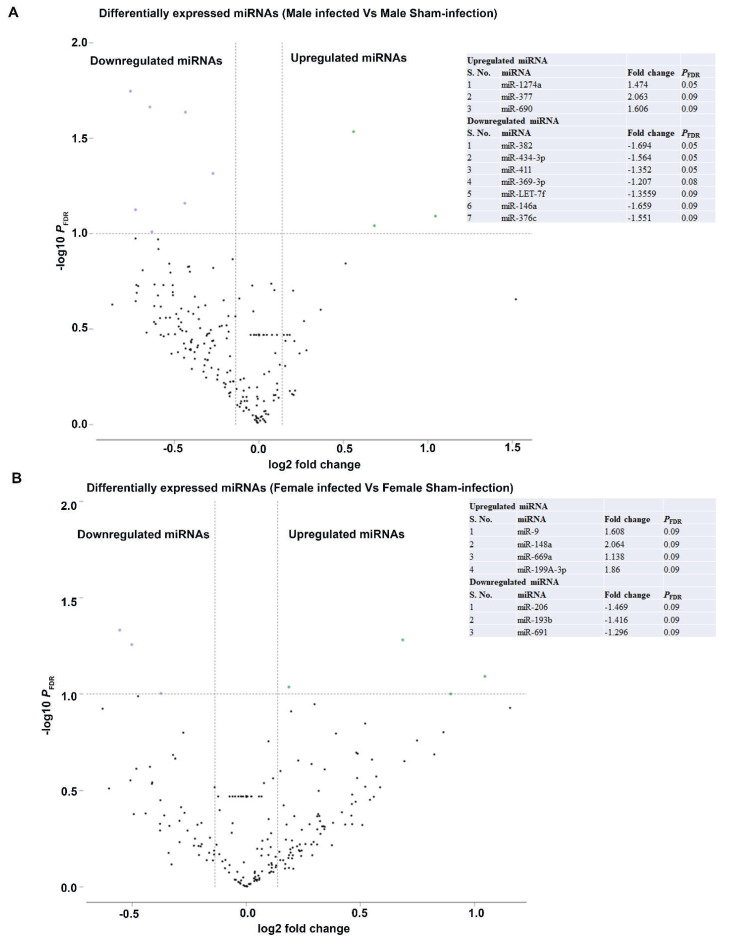
Differentially expressed miRNAs in male and female mice infected with partial human mouth microbes (PAHMM) compared to sham infection. (**A**) The volcano plot depicts the upregulated (green) and downregulated (purple) miRNAs that showed a fold difference of ±1.1 with *p*_FDR_ of < 0.1. The log ratio of the fold change is on the *x*-axis, and the negative log of the *p*-value is on the *y*-axis. The black dots represent the miRNAs that do not pass the filter parameters. Three significant upregulated miRNAs and seven significant downregulated miRNAs are shown in the table. (**B**) The volcano plot depicts the upregulated (green) and downregulated (purple) miRNAs that showed a fold difference of ±1.1 with *p*_FDR_ of < 0.1. The log ratio of the fold change is on the *x*-axis, and the negative log of the *p*_FDR_ is on the *y*-axis. The black dots represent the miRNAs that do not pass the filter parameters. Four upregulated miRNAs and three downregulated miRNAs are shown in the table.

**Figure 4:**
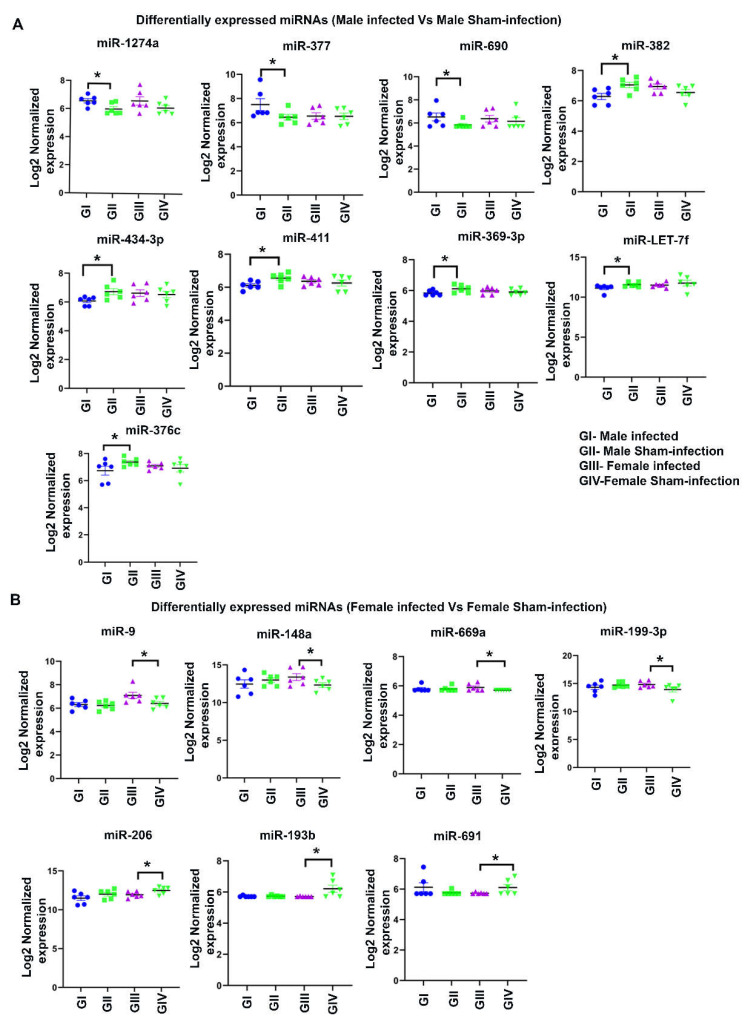
Differentially expressed miRNAs in male and female mice infected with partial human mouth microbes (PAHMM) compared to sham-infected mice. (**A**) Both upregulated miRNAs (miR-1274a, miR-377, and miR-690) and downregulated miRNAs (miR-382, miR-434-3p, miR-411, miR-369-3p, miR-LET-7f, and miR-376c) in male mice infected with PAHMM showed a fold difference of ±1.1 with *p*_FDR_ of < 0.1. The log2-normalized expression of each miRNA is shown on the *y*-axis, and the details of the group are shown on the *x*-axis. (**B**) Both upregulated miRNAs (miR-9, miR-148a, miR-669a, and miR-199-3p) and downregulated miRNAs (miR-206, miR-193b, and miR-691) in female mice infected with PAHMM showed a fold difference of ±1.1 with *p*_FDR_ of < 0.1. The log2-normalized expression of each miRNA is shown on the *y*-axis, and the details of the group are shown on the *x*-axis (* *p*_FDR_ < 0.1; *n* = 6).

nCounter miRNA expression profiling showed 190 DE miRNAs in male infected mice and 188 DE miRNAs in female infected mice (Figure 2C). Among 378 DE miRNAs, miRNAs that showed > 1-fold difference with a *p*-value between 0.9 to 0.0001 were used for data analysis. The number of DE miRNAs in both sexes infected with bacteria was not significantly different. The Venn diagram of the upregulated miRNAs between male and female bacteria-infected mice shows unique upregulated miRNAs in male (34.2%) and female (46.1%) mice. In both sexes of bacteria-infected mice, 19.7% of the miRNAs were commonly upregulated (Figure 2D). Similarly, the Venn diagram of the downregulated miRNAs between male and female bacteria-infected mice shows unique downregulated miRNAs in male (41.3%) and female (32%) mice. In both sexes of bacteria-infected mice, 26.7% of the miRNAs were commonly downregulated (Figure 2E). Further analysis with a higher fold difference from 1.1 to 2.0 and a *p*_FDR_-value of < 0.1 showed highly significant DE of miRNA in both male and female infected mice. The volcano plot depicts three upregulated miRNAs (miR-1274a, miR-377, and miR-690) and seven downregulated miRNAs in male infected mice (miR-382, miR-434-3p, miR-411, miR- miR-369-3p, miR-LET-7f, miR-146a, and miR-376c) (Figure 3A and Figure 4A). Similarly, four upregulated (miR-9, miR-148a, miR-669a, and miR-199a-3p) and three downregulated miRNAs (miR-206, miR-193b, and miR-69) were observed in female infected mice (Figure 3B and Figure 4B). These DE miRNAs were unique; no common miRNAs were found between male and female mice. No significant difference was observed in major inflammatory miRNAs such as miRNA-155, miR-132, or miR-146a expression in bacteria- and sham-infected mice, nor between sexes (Figure S1B).

## 3. Discussion

We investigated the sex-specific levels of the colonization/infection of five bacteria, the microbe-induced IgG immune antibody response, intravascular dissemination, and ABR after infection. All five bacteria were colonized in the gingival surface of the mice, which we confirmed through 16S rRNA gene-specific amplification. As expected, both sexes of mice that were infected by ETSPPI had a significantly higher maxillary and mandibular ABR. This finding provides direct evidence that all mice infected with ETSPPI that mimics PAHMM exhibited periodontitis because ABR is considered one of the outcome measurements of chronic periodontitis. The bone resorption measurement results are consistent with previously published studies in which polybacterial infection in several mouse models (e.g., ApoE^−/−^, itgβ6, TLR2^−/−^, and TLR4^−/−^) induced higher bone resorption [21,23,24,25,26]. No significant difference in ABR was observed between male and female mice infected with ETSPPI.

In contrast, there were sex-specific differences in the genomic DNA of all five bacteria detected in heart tissue. The intravascular dissemination of all five bacteria from gingival tissues to the heart indicates a synergistic interaction of early, intermediate, and late colonizers that adhered to the gingiva after ETSPPI. Furthermore, the observed in vivo male-specific dissemination to the heart directly correlates with the Atherodent clinical trial, in which the association between PD and unstable angina was more common in men, with an average age of 54 years [27]. In addition, a recent study conducted in Spain identified a higher prevalence of cardiovascular disease (CVD) among patients with PD, which was almost double in men [28]. It is important to observe that genomic DNA of all five bacteria was detected in human aortic atherosclerotic plaque and CVD [29]. We previously demonstrated that *P. gingivalis, T. denticola, T. forsythia,* and *F. nucleatum* induced atherosclerotic aortic plaque in an ApoE^−/−^ model of atherosclerosis [25,26]. Further data indicate that *S. gordonii* has a highly invasive potential to disseminate from gingival epithelial tissues to distal organs such as the lungs, kidney, and liver and to gain access to systemic circulation. We also observed a similar dissemination pattern of *S. gordonii* to the heart, lungs, spleen, and kidneys in both APP-transgenic CRND8 (Tg) and wild-type nontransgenic (ntg) mice [30]. In addition, *S. gordonii* is not only a commensal supragingival bacterium but also an opportunistic pathogen, and it can enter the bloodstream, leading to endocarditis [31]. It can also cause apical periodontitis or caries and exists on the heart valves of patients with infective endocarditis [32,33,34,35].

The female-specific intravascular dissemination of the five bacteria to the lungs was surprising. Oral microbiota settling down in the oral microecosystem is known as the main source of the lung microbiome, and it is strongly associated with the occurrence and development of several respiratory diseases (e.g., pneumonia, chronic obstructive pulmonary disease (COPD), lung cancer, cystic fibrosis lung disease, asthma) [36]. In addition, poor oral health and periodontal bacterial dysbiosis (i.e., aspiration of oral microbes and interactions between oral microbes and respiratory pathogens) are related to the risk of multiple respiratory diseases or lung infection. A recent review supports a possible association between poor periodontal health, the frequency of exacerbations, hospitalization, and quality of life in patients with COPD [37].

Significant serum IgG antibody levels of *P. gingivalis*, *T. denticola*, and *T. forsythia* in female mice were observed, whereas in male mice, no significant serum IgG antibody levels were observed for *T. denticola* or *T. forsythia*. These in vivo data clearly suggest that these bacteria can synergistically alter the host’s immune competence with the increased production of virulence factors (i.e., *P. gingivalis* virulence factors decrease the host response) by subverting innate-immune signaling [38]. Thus, a sex-specific difference in the induction of immunity correlates with sex-specific differences in the oral microbiome and the clinical severity and incidence of PD among women and men [15]. High-throughput miRNA profiling documented 190 DE miRNAs in male mandibles and 188 DE miRNAs in female mandibles. The DE miRNAs in male and female mice were unique. Although ABR and IgG antibody levels in both male and female mice were similar, but the miRNA expression profiles in the mandibles of male and female mice were unique (76%), and only < 24% of the miRNAs were common. This suggests that miRNA expression in the ETSPPI model with PAHMM is based on sex as a biological variable. A homogenous sex group generates more consistent data than a mixed-sex one; most of our previous studies used male mice for experimental periodontitis. Our previous study with three major periodontal pathogens, namely *P. gingivalis*, *T. denticola*, and *T. forsythia*, in ApoE^−/−^ male mice showed an enhanced expression of miR-146a in the maxilla and spleen, which was considered a dominant inflammatory miRNA [2]. However, the ETSPPI used in this study with five different bacterial infections did not show the enhanced expression of miR-146a, miR-132, or miR-155. Multiple explanations may have contributed to these different miRNA’s observations, such as the use of a different mouse model, the change in infection schedule, the addition of two bacteria (*S. gordonii* and *F. nucleatum*), the mouse-infected mandible tissue miRNA, and most importantly the use of high-throughput nCounter^®^ miRNA Expression Panels.

We reported three (miR-1274a, miR-377, and miR-690) and four (miR-9, miR-148a, miR-669a, and miR-199A-3p) upregulated miRNAs in male and female mice, respectively. This is the first report to show the presence of these upregulated miRNAs in the PAHMM mouse model. Among the seven upregulated miRNAs, only miR-9 [1,22] and miR-148a [6] have been reported to be upregulated in inflamed gingival tissues in humans. The other miRNAs (miR-199a-3p, miR-1247a, miR-377, miR-690, and miR-669a) identified in this study have not been previously evaluated in patients with periodontitis. Hence, further studies using high-throughput techniques are warranted to identify global miRNAs in inflamed human gingival tissues. miR-9 has been shown to classically activate macrophages and various pro-inflammatory molecules, and it plays an important role in regulating the inflammatory response [39]. On the other hand, miR-148a is also closely associated with the MAPK signaling pathway and the NF-kB pathway, an important pathway in inflammation and the immune system [40,41]. Hence, our observation of a higher expression of miR-9 and miR-148a in the ETSPPI mouse model supports the concept that these miRNAs are important regulators of an inflammatory response in experimental PD. Furthermore, miR-9 and miR-148a have been closely associated with breast, gastric, lung, and colorectal cancers in humans [42,43,44,45,46,47,48].

miR-199a-3p has been reported to be a tumor-suppressor miRNA in lung cancer proliferation [49], and miR-1274a has been reported as an oncogene and prognostic marker for colon cancer [50] and non-small-cell lung cancer [51]. Further studies with miRNA knockout mice may reveal other possible functions of miR-199a-3p and miR-1274a in experimental periodontitis. The male-specific miRNA-377, which was significantly (2-fold) upregulated only in male mice, also regulates cerebral inflammation and angiogenesis in ischemic stroke [52]. Interestingly, several observational, cross-sectional [53,54], case-control [55,56], and cohort studies [57,58] determined an association between PD and stroke incidence that strongly supports miRNA-377 as a direct link between microbe-induced gingival and cerebral inflammation. A strong positive relationship was found between miR-377 upregulation in males and the genomic dissemination of all five bacteria to male heart tissue. Furthermore, miR-377 has been linked with other inflammatory diseases, such as atherosclerosis [59] and Sjogren’s syndrome [60], indicating a strong miRNA link between atherosclerosis and periodontitis. miRNA-690 has been implicated in inflammation and endoplasmic reticulum stress in obesity [61]. Further studies are warranted to determine whether miR-377 and miRNA-690 play an important role in proinflammatory cytokine responses in periodontal bacteria–mediated inflammation. A detailed comparison analysis of the presence of important miRNAs identified in this study in human periodontitis and other diseases is given in Table 4.

## 4. Materials and Methods

### 4.1. Bacterial Strains, Growth Conditions, and Polybacterial Preparations

*S. gordonii* DL1, *F. nucleatum* ATCC 49256, *P. gingivalis* 381, *T. denticola* ATCC 35405, and *T. forsythia* ATCC 43037 were used in this study. *P. gingivalis* 381, *S. gordonii* DL1, and *F. nucleatum* ATCC 49256 were grown in Brucella blood agar plates supplemented with hemin and vitamin K (Hardy Diagnostics, Santa Maria, CA, USA). The oral spirochete *T. denticola* ATCC 35405 strain was grown in GM-1 broth [2,21], and *T. forsythia* ATCC 49307 was grown in TSB broth supplemented with N-acetyl muramic acid (5 mg/mL) and hemin (1 mg/mL) [21,63]. All the bacteria were cultured and harvested in a Coy anaerobic chamber at 37 °C for 2 to 3 days as previously described [24,25,30,63]. *P. gingivalis*, *S. gordonii,* and *F. nucleatum* were harvested from the media plates by using a sterile cotton tip applicator. The log-phase culture of *T. denticola* and *T. forsythia* was harvested by centrifugation at 8000 rpm for 10 min, and the respective pellets were washed with 5 mL of phosphate-buffered saline (PBS) followed by suspension in 1 mL of reduced transport fluid (RTF). An equal volume of *P. gingivalis* and *T. denticola* in RTF was taken in round bottom culture tubes and incubated for 5 min inside an anaerobic chamber (Hausser Scientific Company, Horsham, PA, USA). After incubation, *T. forsythia* was added to the culture tubes, and it was allowed to interact for an additional 5 min. Bacterial cells were counted using a Petroff–Hausser bacterial counting chamber. The three bacterial mixtures (10^8^ cells each) were then mixed with an equal volume of 6% carboxymethylcellulose (CMC), and polybacterial infection was performed. An equal volume of RTF and 6% CMC was used as a vehicle control for sham-infected mice.

### 4.2. Ecological Time-Sequential Polybacterial Periodontal Infection in C57BL/6J Mice

Subgingival microbial dysbiosis in the periodontium leads to the progression of inflammatory periodontitis. Periodontal microbes colonize in supragingival and subgingival regions and activate various proinflammatory cytokines and chemokines, leading to the breakdown of connective tissues and this causes ABR. Understanding the host–pathogen synergistic interaction during the progression of inflammation and identifying the key miRNAs involved in inflammatory reactions is important for designing prognostic/diagnostic markers for inflammatory PD. Because the prevalence of chronic periodontitis differs between men and women, it is imperative to identify whether miRNA expression also varies between sexes in experimental PD. To partially mimic the normal bacterial colonization that occurs in the human oral cavity from youth to adulthood, we used an ETSPPI model in C57BL/6J mice.

Both female and male C57BL/6J mice were purchased from Jackson Laboratories (Bar Harbor, ME, USA). Mice were housed in microisolator cages and fed standard chow and sterile water ad libitum. All mouse cages were maintained at 25 °C with alternating 12-h periods of light and dark. To avoid cross-contamination, sham-infected mice were maintained in separate rooms. All animal procedures were done in accordance with the approved protocol guidelines from the University of Florida Institutional Animal Care and Use Committee (IACUC protocol #201910761). Nine-week-old male and female mice were randomly divided into groups for polybacterial infection (*n* = 10) and sham infection (*n* = 10) (Table 1). After acclimation, mice were initially administrated kanamycin (500 mg/kg) in sterile water for 3 d followed by swabbing with 0.12% chlorhexidine gluconate (Peridex: 3M ESPE Dental Products, St. Paul, MN, USA) mouth rinse on day 5. This initial treatment suppresses existing oral bacteria and facilitates the bacterial colonization with *S. gordonii*, *F. nucleatum*, *P. gingivalis*, *T. denticola*, and *T. forsythia*. To mimic the human ecological bacterial colonization, ETSPPI was performed. Polybacterial-infected mice were first infected with *S. gordonii* (an early colonizer; 2.5 × 10^8^ cells) for 2 infection cycles (1 infection cycle constitutes 4 consecutive infection days in a week), followed by infection with *F. nucleatum* (an intermediate colonizer, bridging bacterium; 2.5 × 10^8^ cells) for 2 infection cycles. Finally, this was followed by infection with *P. gingivalis*, *T. denticola,* and *T. forsythia* (late colonizers; 2.5 × 10^8^ cells each) for 6 infection cycles. Because PD is associated with polybacterial synergy and dysbiosis of the oral bacterium, ETSPPI in the current study with PAHMM (early, intermediate, and late bacterial colonizers) in mice partially mimics the ecological bacterial shifts from youth to adulthood in humans.

Sequential polybacterial infection was performed as illustrated in Figure 1A. Mouse gingival surfaces were swabbed with a sterile cotton swab every alternate infection week, and 16S rRNA gene-specific PCR for specific bacteria was performed to monitor bacterial adhesion/colonization and the subsequent invasion of the gingival epithelium. At the end of the 20-week infection period, mice were euthanized. Right maxilla and mandibles were collected for bone morphometry analysis. Left maxilla and mandibles were stored in RNAlater (Invitrogen, Waltham, MA, USA). Left mandibles (periodontium) with three molars were used for Nanostring analysis. Blood was drawn through cardiac puncture, and serum was isolated and stored in a −20 °C freezer for measuring IgG antibodies against specific bacteria.

### 4.3. Bacterial Genomic DNA Detection in Gingival Plaque Using PCR

After 4 days of infection, gingival plaque samples from the gingival surface were collected from all infected and sham-infected mice by using sterile cotton swabs. Bacterial genomic DNA was detected using 16S rRNA gene species-specific primers of the bacteria using Phusion High Fidelity Master Mix from New England Biolabs (NEB, Ipswich, MA, USA) as described previously [21,25,26,64]. Briefly, colony PCR was performed with a Bio-Rad Thermal Cycler (Bio-Rad, Hercules, CA, USA) using *S. gordonii*-specific 16S rRNA gene-specific forward primer 5′-GTAGCTTGCTACACCATAGA-3′, reverse primer 5′-CTCACACCCGTTCTTCTCTT-3′; *F. nucleatum*-specific 16S rRNA gene-specific forward primer 5′-TAAAGCGCGTCTAGGTGGTT-3′, reverse primer 5′-ACAGCTTTGCGACTCTCTGT-3′; *P. gingivalis*-specific 16S rRNA gene-specific forward primer 5′-GGTAAGTCAGCGGTGAAACC-3′, reverse primer 5′-ACGTCATCCACCCTTCCTC-3′; *T. denticola*-specific 16S rRNA gene-specific forward primer 5′-TAATACCGAATGTGCTCATTTACAT-3′, reverse primer 5′-CTGCCATATCTCTATGTCATTGCTCTT-3′; *T. forsythia*-specific 16S rRNA gene-specific forward primer 5′-AAAACAGGGGTTCCGCATGG-3′, and reverse primer 5′-TTCACCGCGGACTTAACAGC-3′. All these primers amplify partial 16S rRNA gene sequences of the respective bacterium, and we confirmed their specificity by NCBI Primer BLAST followed by in silico analysis of the bacterial genomes. Primers for *S. gordonii* were designed after multiple sequence alignment using Clustal Omega. Genomic DNA extracted from the respective bacterium was used as a template for positive control, and samples with no bacterial DNA were used as a negative control. PCR products were run on 1% agarose gel electrophoresis and visualized under UVP GelStudio touch Imaging System (Analytik Jena US LLC, CA, USA).

### 4.4. Bacterial Systemic Dissemination to Distal Organs

Genomic DNA from an aliquot of the heart comprising the right atrium and right ventricle and other organs such as the lungs, kidney, and liver was extracted following a standard protocol described in the Qiagen Dneasy Blood and Tissue kit (Qiagen, Germantown, MD, USA). 16S rRNA gene-specific PCR was performed for the respective bacterium using specific bacterial primers as described [21,25,26,64].

### 4.5. Antibacterial Serum Immunoglobulin G Antibody Analysis by Enzyme-Linked Immunosorbent Assay

A serum IgG antibody titer against each of the five bacterial species from all the mice in the four groups was analyzed by using enzyme-linked immunosorbent assay (ELISA) as described previously [21,25,26,64]. Whole cell FK bacteria such as *S. gordonii* (1:60 dilution), *F. nucleatum* (1:50), *P. gingivalis* (1:150), *T. denticola* (1:30), and *T. forsythia* (1:160) were used as coating antigen, and 100 µL of 1:100 diluted serum of each mouse in triplicate was added to the wells. The plates were incubated for 2 h. One hundred microliters of goat anti-mouse IgG alkaline phosphatase (Sigma Aldrich, St. Louis, MO, USA) was added, incubated, and developed with 200 µL of p-nitrophenylphosphate for 15 min, and color development was stopped using 3M NaOH. The plates were read at OD 405 nm and analyzed using Gen5 software in Epoch Microplate Spectrophotometer (BioTek, Winooski, VT, USA). IgG serum antibody concentrations were determined by using the gravimetric standard curve (Sigma Aldrich).

### 4.6. Alveolar Bone Resorption by Morphometry

The horizontal ABR area of infected male and female mice was measured by histomorphometry as described previously [21,25,26,64]. The right mandibles and maxilla were autoclaved, and the defleshed jaws were immersed in 3% hydrogen peroxide for 30 min and air-dried. Two-dimensional molar teeth images were captured using a stereo dissecting microscope (Stereo Discovery V8, Carl Zeiss Microimaging, Inc, Thornwood, NY, USA). The area between the cemento-enamel junction to the alveolar bone crest of the buccal and the palatal surfaces of the maxillary jaws was measured by using the line tool (AxioVision LE 29A software version 4.6.3, Thornwood, NY, USA). Two examiners blinded to the study mice groups measured the ABR.

### 4.7. Extraction and Quantification of RNA for Nanostring Analysis

Mandibles (left; *n* = 6) from male and female mice (four groups) were taken for Nanostring analysis. The total RNA from each mandible was extracted based on the protocol described in the mirVana miRNA isolation kit (Ambion, Austin, TX, USA). Initially, mandibles from each mouse were entirely homogenized using the handheld rotor-stator homogenizer with sterile individual TissueRuptor disposable probes (Qiagen; Germantown, MD, USA) for each sample. After homogenization, each sample was lysed in a denaturing lysis solution that stabilized RNAs and inactivated RNases. The samples were subjected to an acid-phenol:chloroform extraction that removed all the cellular components such as protein, DNA, and other cellular products. The aqueous phase was removed, transferred to the fresh nuclease-free microcentrifuge tube, and 1.25 volume of 100% ethanol was added. This mixture was transferred to the filter cartridge placed into the collection tube and centrifuged for 15 s at 10,000× *g*. The filter cartridge was washed with 700 µL of wash solution-1 followed by washing with wash solution-2/3. After washing, the total RNA was eluted from the filter cartridge with 100 µL of nuclease-free water. RNA yield and purity were determined using a Take3 micro-volume plate in Epoch Microplate Spectrophotometer (BioTek), and quantification was performed in technical duplicates for each sample.

### 4.8. nCounter^®^ miRNA Expression Profiling

We used high-throughput nCounter^®^ miRNA Expression Panels (Nanostring Technologies, Seattle, WA, USA) to effectively identify the DE of miRNA in both sexes. Nanostring nCounter^®^ analysis can identify 577 miRNAs in any sample, and by using molecular barcodes, it can detect even a low number of miRNAs without the need for reverse transcription or amplification.

miRNA expression profiling was performed using the Nanostring nCounter^®^ Mouse miRNA Assay kit v1.5 (Nanostring Technologies). This assay is a highly sensitive multiplexed method that detects miRNAs using molecular barcodes called nCounter reporter probes without the need for reverse transcription. Sample preparation involved annealing, ligation, and purification. These steps were carried out based on the experimental procedure described in the nCounter^®^ miRNA assay panel kit. Briefly, the annealing master mix was prepared by combining 13 µL of annealing buffer, 26 µL of nCounter miRNA Tag reagent, and 6.5 of diluted (1:500) miRNA assay controls. Next, 3.5 µL of the annealing master mix was aliquoted into each tube of the strip tube and 100 ng of the total RNA from six left mandibles from each group (maximum 3-µL volume) with 260/280 and 260/230 ratios of greater than 2 was added to the respective tubes. The strip tube was placed in the Thermal Cycler with the following conditions: 94 °C for 1 min, 65 °C for 1 min, 45 °C for 1 min, and 48 °C for hold.

After annealing, 2.5 µL of the ligation master mix (19.5 µL of polyethylene glycol (PEG) and 13 µL of ligation buffer) was added to all the tubes in the strip tube. The strip tube was incubated at 48 °C for 5 min, followed by the addition of 1 µL of ligase into each tube without removing the strip tubes from the Thermal Cycler. Ligation was performed with the following conditions: 48 °C for 3 min, 47 °C for 3 min, 46 °C for 3 min, 45 °C for 3 min, 65 °C for 10 min, and 4 °C for hold. To remove the unligated tags, a purification step was performed after adding 1 µL of ligation cleanup enzyme to all the tubes and incubating the tubes at 37 °C for 1 h, 70 °C for 10 min, and 4 °C for hold. Forty microliters of RNase-free water were added to each tube in the strip tube and the sample was ready for hybridization with the nCounter reporter and capture probes. After denaturation at 85 °C for 5 min, a 5-µL aliquot from the miRNA sample preparation tube was taken along with the 10 µL of miRNA reporter code, 10 µL of hybridization buffer, and 5 µL miRNA capture probe. The strip tubes were incubated at 65 °C for 18 h in the Thermal cycler, and the samples were immediately processed for post-hybridization with the nCounter analysis system at the Molecular Pathology Core at the University of Florida.

The nCounter^®^ Mouse miRNA Assay kit v1.5 provided six positive hybridization controls and eight negative control probes to monitor hybridization efficiency. All components and reagents needed for sample preparation at the reparation station were taken from the nCounter master kit (Nanostring Technologies). Twelve samples per cartridge were processed in a single run, which took 3 h. This was followed by digital analysis, which involved the transfer of the cartridge to the multichannel epifluorescence digital analyzer. A cartridge definition file with a maximum fields of view (FOV) count of 555 per flow cell was taken for digital analysis. The number of images taken per scan corresponded to the number of immobilized reporter probes on the cartridge. A separate Reporter Code Count (RCC) file for each sample containing the count for each probe was downloaded and used for data analysis.

### 4.9. Nanostring Data Analysis

Initial data analysis was performed using nSolver 4.0. Initially, RCC files were imported into nSolver and imaging quality control (QC), binding density QC, positive control linearity QC, and positive control limit of detection QC were carried out as recommended in the system QC parameters. The lanes were flagged when the percent FOV registration was less than 75% for imaging QC. Binding density was outside the range from 0.1 to 2.25 for binding density QC, and the positive control R^2^ value was less than 0.95. All 24 samples passed the QC, and no flag lanes were observed. Raw data were generated after passing the QC. To reduce the background signal/noise, the background threshold count value was set to 52 and calculated by taking an average of eight negative control probe counts from all 24 samples. Codeset content normalization parameters were chosen, and the normalization was performed based on the top 100 miRNA genes expressed. The normalized factor was calculated based on the geometric mean values of the miRNA genes expressed in each sample. All the normalized data were further analyzed using ROSALIND^®^ with a hyperscale architecture developed by ROSALIND Inc. (San Diego, CA, USA). Fold changes in the genes were calculated based on the ratio of the difference in the means of the log-transformed normalized data to the square root of the sum of the variances of the samples in the two groups. The limma R library [65] was used to calculate fold changes. Clustering of miRNA for the final heatmap of DE miRNA was done using the Partitioning Around Medoids method with the fpc R library.

### 4.10. Statistical Analysis

One-way ANOVA with Dunnett’s multiple comparisons was performed for multiple group comparisons to determine the statistical significance, using the statistical software Prism 9.2.0 (GraphPad Software, San Diego, CA, USA). All the data in graphs are presented as mean ± SEM. *p*_FDR_-value < 0.1 was considered statistically significant, and the *p*_FDR_-values were calculated based on the Benjamini–Hochberg procedure [66]. The Mann–Whitney test was used for IgG antibody analysis. Two-tailed *t*-testing was performed on the log-transformed normalized data that assumed unequal variance to identify the differential gene expression. The distribution of the t-statistics was calculated using the Welch–Satterthwaite equation for the degrees of freedom to estimate the 95% confidence intervals for the identified DE of miRNA between groups. Fisher’s exact tests were performed to compare the frequencies of bacterial 16S rRNA gene detection in the heart and lungs of experimental male and female mice. Analysis was performed using STATA software version 14.0 (StataCorp, College Station, TX, USA). *p* < 0.05 was considered significant.

## 5. Conclusions

In conclusion, the present study is the first to provide evidence of an efficient oral microbial colonization (PAHMM) and novel infection (ETSPPI) PD mouse model that partially mimics human ecological colonization involving five oral bacteria. Furthermore, we found sex-specific DE of miRNA in male (miR-1274a, miR-377, miR-690, miR-382, miR-434-3p, miR-411, miR-369-3p, miR-LET-7f, and miR-376c) and female (miR-9, miR-148a, miR-669a, miR-199-3p, miR-206, miR-193b, and miR-691) mouse mandibles in ETSPPI. Although this study does not provide insight into the putative targets of the reported miRNAs, it is the first study to use high-throughput global Nanostring analysis to identify previously unreported miRNAs (miR-1274a, miR-377, miR-690, miR-382, miR-434-3p, miR-411, miR-369-3p, miR-LET-7f, miR-376c, miR-9, miR-148a, miR-669a, miR-199-3p, miR-206, miR-193b, and miR-691) in novel ETSPPI in a PAHMM mouse model. These gingival inflammatory miRNAs can be considered potential biomarkers of periodontitis and potentially other connected systemic diseases (e.g., atherosclerosis, stroke, and diabetes). Future mechanistic studies that investigate the expression, biological roles, and underlying mechanisms of miRNAs in periodontitis may provide diagnostic biomarkers and valuable therapeutic targets in the treatment of chronic periodontitis.

## Figures and Tables

**Table 1:** Details of experimental and control groups (*n* = 39 mice).

Group	Bacteria and Oral Infection	Sex	Number of Mice
I	C57BL/6J mice—ETSPPI (*Sg* + *Fn* + *Pg/Td/Tf*)	Male	10
II	C57BL/6J mice—sham-infection (Vehicle control)	Male	10
III	C57BL/6J mice—ETSPPI (*Sg* + *Fn* + *Pg/Td/Tf*)	Female	10
IV	C57BL/6J mice—sham-infection (vehicle control)	Female	9

*Sg*—*Streptococcus gordonii* DL-1; *Fn*—*Fusobacterium nucleatum* ATCC 49256; *Pg*—*Porphyromonas gingivalis* FDC 381; *Td*—*Treponema denticola* ATCC 35405; *Tf*—*Tannerella forsythia* ATCC 43037; ETSPP—ecological time-sequential polybacterial periodontal infection.

**Table 2:** Gingival plaque samples positive for bacterial genomic DNA by PCR.

Group/Mice/Infection	Positive Gingival Plaque Samples (*n* = 10 Mice)				
	2 weeks (*Sg*)	6 weeks (*Fn*)	8 weeks (*Fn*)	10 weeks *Pg/Td/Tf*	14 weeks *Pg/Td/Tf*
Group I/Male C57BL/6J mice/ETSPPI	10	5	10	10/5/5	10/6/10
Group II/Male C57BL/6J mice/Sham-infection	0	0	NC	0/0/0	NC
Group III/Female C57BL/6J mice/ETSPPI	10	7	10	8/7/6	10/9/10
Group IV/Female C57BL/6J mice/Sham-infection	0	0	NC	0/0/0	NC

Total numbers of gingival plaque samples that were collected after infections (2, 6, 8, 10, and 14 weeks): *S. gordonii* DL1 infection (week 2), *F. nucleatum* ATCC 49256 infection (weeks 6 and 8), and polybacterial infection (*P. gingivalis* FDC 381, *T. denticola* ATCC 35405, and *T. forsythia* ATCC 43037; weeks 10 and 14). Positive infections were determined by PCR analysis. NC—not collected; ETSPPI—ecological time-sequential polybacterial periodontal infection.

**Table 3:** Distribution of genomic DNA of periodontal bacteria to distal organs.

Positive Systemic Tissue Specimens (*n* = 10)					
Bacterial Infection	Sex (Male/Female)	Heart	Lungs	Kidney	Liver
*S. gordonii*	M	9	0	6	9
	F	4	7 *	4	9
*F. nucleatum*	M	9*	0	0	0
	F	3	9 *	0	0
*P. gingivalis*	M	4	1	0	0
	F	2	8 *	0	0
*T. denticola*	M	10 *	0	0	0
	F	0	1	0	0
*T. forsythia*	M	9	0	0	0
	F	5	9 *	0	0

To analyze the systemic bacterial infection, total genomic DNA from an aliquot of the mouse heart (comprising the right atrium and right ventricle), lungs, kidney, and liver was extracted. The extracted genomic DNA was examined for the presence of bacterial DNA through the respective bacterium-specific 16S rRNA gene primers. The dissemination of all five bacteria from the oral gingival surface to the heart represents their invasive potential. Intravascular dissemination to heart tissue was higher in males than females, which was significant for *F. nucleatum* and *T. denticola* (* *p* < 0.05, Fisher’s exact test). There was significantly higher dissemination of all bacteria except *T. denticola* in the lungs of females compared to males (* *p* < 0.05, Fisher’s exact test).

**Table 4:** Comparison of the presence of the dominant miRNAs in this study in human periodontitis and other diseases.

This Study	Human PD	Other Diseases
**Female Mice**		
miR-9	Inflamed gingival tissues [1,22]	Gene regulators in many human cancers (e.g., breast, colorectal, hepatocellular, non-small-cell lung) [42,43,44,45]
miR-148a	Inflamed gingival tissues [6]	Gene regulators in many human cancers (e.g., breast, hepatocellular, lung) [46,47,48]
miR-669a	Not reported	Not reported
miR-199a-3p	Not reported	Tumor-suppressor miRNA in lung cancer [49]
**Male mice**		
miR-1274a	Not reported	Oncogene and prognostic marker for colon cancer [35] Non-small-cell lung cancer [51]
miR-377	Not reported	Cerebral inflammation and angiogenesis in ischemic stroke animal models [52] Atherosclerosis [62] and Sjogren’s syndrome [60]
miR-690	Not reported	Inflammation and endoplasmic reticulum stress in obese animal models [61]

## Data Availability

The raw data and other related data in the manuscript are available from the corresponding author, L.K., upon reasonable request.

## Supplementary Materials

The following supporting information can be downloaded at: https://www.mdpi.com/article/10.3390/ijms23095107/s1.
