# Supplementary material for: Global Noncoding microRNA Profiling in Mice Infected with Partial Human Mouth Microbes (PAHMM) Using an Ecological Time-Sequential Polybacterial Periodontal Infection (ETSPPI) Model Reveals Sex-Specific Differential microRNA Expression
Source: Int J Mol Sci. 2022 May 4;23(9):5107. doi: 10.3390/ijms23095107 (PMC9105503; doi:10.3390/ijms23095107)
Supplement: Supplementary file 1 [file ijms-23-05107-s001.zip › ijms-1683022-supplementary.pdf]

International Journal of *Molecular Sciences*

MDPI

Article

Global noncoding microRNA profiling in mice infected with partial human mouth microbes (PAHMM) using an ecological time-sequential polybacterial periodontal infection (ETSPPI) model reveal sex-specific differential microRNA expression

Chairmandurai Aravindraja [1], Matteen R. Kashef [1], Krishna Mukesh Vekariya [1], Ravi K. Ghanta [2], Shama Karanth [3], Edward K. L. Chan [4] and Lakshmyya Kesavalu [1,4*]

Supplemental information

Figure S1.

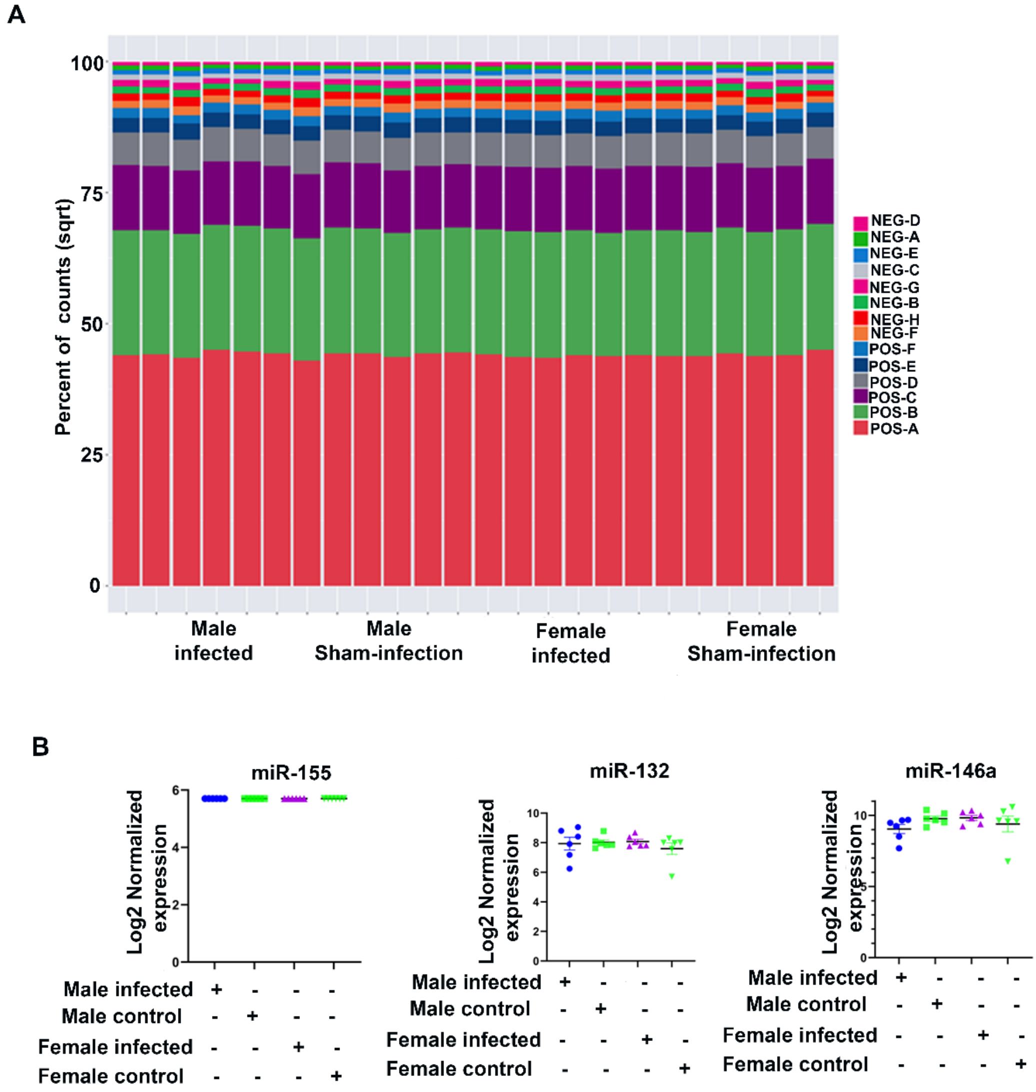

Figure S1. NanoString control plot and dominant miRNA expression in PAHMM using ETSPPI mouse model. A. This plot shows the expression levels of spike-ins, negative controls and

housekeeping genes. Each column has been summed to 100%. The expression levels of the controls were more or less similar in all the samples that showed the ligation and hybridization was successful in all the samples during the sample preparation. B. Expression pattern of dominant miRNAs such as miR-155, miR-132 and miR-146a was not observed to be significant in ETSPPI mouse model.

Table S1. RNA quantification of mandibles for NanoString analysis

Sample	260/280	260/230	ng/μl
GI-1	2.033	2.089	188.30
GI-2	2.085	2.039	152.05
GI-3	2.117	2.011	144.95
GI-4	2.056	2.425	224.11
GI-5	2.107	2.153	208.44
GI-6	2.074	2.179	344.48
GII-1	2.114	2.125	533.32
GII-2	2.069	2.091	999.91
GII-3	2.1	2.088	564.03
GII-4	2.12	2.243	258.54
GII-5	2.084	2.371	310.25
GII-6	2.099	2.235	591.70
GIII-1	2.078	2.0	435.20
GIII-2	2.086	2.017	237.49
GIII-3	2.108	2.228	271.41
GIII-4	2.082	2.241	282.74
GIII-5	2.076	2.153	230.39
GIII-6	2.051	2.219	285.48
GIV-1	2.097	2.166	378.13
GIV-2	2.074	2.404	423.07
GIV-3	2.077	2.44	633.68
GIV-4	2.045	2.563	729.69
GIV-5	2.098	2.339	521.42
GIV-6	2.02	1.993	613.68

The quality of total RNA extracted from each mandible was high and 100 ng of RNA was taken for Nanostring analysis.
